# Poliomyelitis in Bristol, 1947

**Published:** 1947

**Authors:** James Macrae

**Affiliations:** Deputy Medical Superintendent, Ham Green Hospital and Sanatorium, Bristol


### POLIOMYELITIS IN BRISTOL, 1947.

BY

JAMES MACRAE, M.D., F.R.F.P.S., D.P.H.

*Deputy Medical Superintendent, Ham Green Hospital and Sanatorium, Bristol.*

DURING the warm dry summer and autumn of 1947 the prevalence of acute anterior poliomyelitis increased in Britain to an extent never before recorded. Cases were widespread but unevenly distributed over the country; and some areas, notably the mountains of Wales and Scotland, showed no epidemic incidence of the disease. The greatest number of cases occurred in August, and the curve was still high above normal in November (see Table I). The Bristol district had a share of this disease, and many of the cases were admitted to Ham Green Hospital.

Infantile paralysis holds an exalted place in the popular imagination and in 1947 journalism did not fail to make it the most talked of, and probably the most dreaded, disease in Britain. As a result the disease received recognition and attention somewhat out of proportion to its importance, even in its epidemic phase. It is necessary to view poliomyelitis in its proper perspective relevant to other diseases.

Within the period February 8th–December 1st 1947, 102 cases of poliomyelitis were notified to the Public Health Department of the City of Bristol and of these, 95 were admitted to Ham Green Hospital: eighteen proved to be true cases. To these must be added four patients found to be suffering from poliomyelitis although admitted as some other disease.

TABLE I. POLIOMYELITIS CASES: BRITAIN, 1946; 1947; BRISTOL, 1947.

| | April | May | June | July | August | Sept. | Oct. | Nov. |
|---|---|---|---|---|---|---|---|---|
| Britain 1946* | 21 | 48 | 47 | 53 | 140 | 115 | 110 | 76 |
| Britain 1947* | 34 | 70 | 176 | 814 | 3,874 | 2,948 | 1,663 | 936 |
| Bristol 1947 | — | 1 | — | 2 | 8 | 5 | 3 | 3 |

**B.M.J.*, 1947—Epidemiology section.

In the past, poliomyelitis has been reported in Bristol in comparable numbers in the years 1921 (25 cases), 1922 (15 cases), 1923 (11 cases), 1926 (10 cases) and 1929 (18 cases). During recent

P

years, the incidence has been 1943 (1 case), 1944 (2 cases), 1945 (5 cases) and 1946 (7 cases).

It will be seen that the available local statistics of the 1947 epidemic do not command respect as a major epidemiological problem. However, these are merely the known figures : they do not take account of missed cases, of abortive attacks, or the extent of the carrier condition, and therefore give little information concerning the spread of the virus of poliomyelitis. Indeed, one of the main difficulties experienced in handling this disease is the lack of confirmatory evidence of the presence of infection, open or cryptic.

In present circumstances " poliomyelitis " is still a diagnosis based on clinical evidence, and diagnostic criteria will necessarily vary from clinician to clinician. Frankly paralytic cases are easy to diagnose ; difficulties arise with patients either in the preparalytic stage, or suffering from the disease but with no paralysis. At Ham Green Hospital it is freely admitted that positive diagnosis may be impossible in some such cases.

In Bristol cases developed in 1947 without any significant distribution throughout the City, and the disease appeared irrespective of work or social conditions. At least two patients were infected outside Bristol.

Of the twenty-two cases regarded as true infections no case appeared to have been a direct infective agent in regard to any of the others. One case seemed to have acquired her infection from a common source, in that a friend developed poliomyelitis at the same time. That patient died in Ham Green Hospital and her friend died in Scotland. The following table sets out the age, sex and severity of illness of the cases.

TABLE II. AGE—SEX—SEVERITY.

| Sex | *Severity | 0–5 | 6–10 | 11–15 | 16–20 | 21–25 | 26–30 | 31–35 | Total |
|---|---|---|---|---|---|---|---|---|---|
| MALE .. | A | 1 | 1 | 2 | 2 | — | — | — | 6 |
| | B | 2 | — | 1 | — | 1 | — | 1 | 5 |
| | C | — | — | — | — | 1 | — | — | 1 |
| FEMALE .. | A | 2 | 3 | — | — | — | 1 | — | 6 |
| | B | 1 | — | — | — | — | — | — | 1 |
| | C | — | — | — | — | — | 3 | — | 3 |

*—A. Transient or no paralysis.
B. Definite paralysis likely to clear up without permanent damage.
C—Paralysis likely to cause permanent damage.

The distribution of paralysis varied from case to case and was sometimes difficult to demonstrate as minor degrees of paresis :

nineteen patients were affected to a more or less extent, and of the mild or moderate degrees of paresis seven were affections of the leg muscles, two were facial paralyses and one was a palatal paralysis.

Severe paralysis occurred in four cases with distribution thus :—

**Case** (*a*), male, 24. Complete paralysis of legs and arms, buttocks, erector spinae and shoulders : retention of urine.

**Case** (*b*), female, 26. Complete paralysis of legs ; partial paralysis of arms and shoulders, paralysis of muscles of respiration including practically the whole of the diaphragm : Died.

**Case** (*c*), female, 29. Almost complete paralysis of both legs ; erector spinae and recti abdominis : retention of urine.

**Case** (*d*), female, 26. Paralysis of pharynx, larynx, palate, and left dome of the diaphragm.

In the past most sporadic cases of poliomyelitis were seen in hospital when they had demonstrable paralysis. During the recent epidemic it was interesting to see some cases in the preparalytic stage. An attempt was made to correlate these two clinical phases, with a view to more accurate assessment of the non-paralytic variety of the disease.

*The Paralytic Phase.* Cases in this phase were admitted still suffering from some of the preparalytic symptoms, evidenced chiefly as nuchal rigidity, headache, spinal rigidity, muscular pains, cramps or pyrexia. The fourteen patients admitted in this stage had been ill for from two to six days previously, and had commenced paralysis between the 1st and 5th day of the disease.

Lumbar puncture was performed on admission and a reaction was noted consisting of increases of cells and protein. The cells appeared as approximately 40 per cent. polymorphonuclears and 60 per cent. lymphocytes, and the numbers varied from 30 to 350 per c.mm. In no other particular were the cerebro-spinal fluids abnormal. The fluid pressures were usually in the upper limits of normal.

Early in this stage patients showed moderate leucocytosis, without gross alteration of the differential white cell picture. There was lower-motor-neurone or bulbar paralysis without sensory loss. Pains in the muscles, especially of the calves and back, remained for several days, and cramps tended to occur at times during the first two weeks.

The patients were all mentally acute ; no patient showed depressive symptoms even in the face of extensive paralysis. On the average, pyrexia subsided four days after paralysis, but some cases showed elevated temperatures for as long as seven days after paresis. Two patients had no pyrexia while in hospital.

*The Preparalytic Phase.*—Patients admitted in this stage of the disease exhibited clinical features remarkably like acute meningitis in various stages of severity. Before admission there had been a prodromal period of malaise lasting from three to ten days, succeeded by a fairly acute onset of headache, vomiting, nuchal rigidity, back pains, pains in the limbs and pyrexia. Three of these cases appeared to be quite toxic on admission ; the others less toxic than their symptoms would have suggested. There was a moderate leucocytosis. Examination of the cerebro-spinal fluid discounted a diagnosis of meningitis, and showed the reaction so commonly noted in the paralytic cases. Tendon reflexes tended to be exaggerated and in two instances knee and ankle clonus was noted.

A diagnosis of poliomyelitis was confirmed by the onset of paralysis in five such cases ; three others developed no paralysis and were considered abortive cases of the disease.

It was remarkable that four of the preparalytic cases developed paralysis as late as the 9th, 13th, 15th and 11th day of disease. The severity of the preparalytic symptoms did not necessarily indicate severe degrees of subsequent paralysis.

*Disposal of Cases.*—Paralysis being established, cases were referred to the judgment of the orthopaedic surgeon, who supervised all future physiotherapy. Fourteen cases received physiotherapy in Ham Green Hospital and of these eight were transferred to Winford Orthopaedic Hospital for further treatment. There being no specific treatment no attempt was made to influence the progress of the disease, apart from nursing, adequate diet, and analgesics. It was found that pethidene was particularly useful in controlling the muscle pains and cramps, which tended to make life miserable for the patient.

Poliomyelitis was regarded as an infectious disease, having respiratory and faecal routes of infection, and the patients were isolated for a period of not less than three weeks. In this connection a nursing orderly in Ham Green Hospital developed an abortive attack of poliomyelitis. She lived out of Hospital and had come in contact with only one case of poliomyelitis in the wards.

Eleven cases were discharged to their own homes ; one patient died, and two are still in hospital. Several of the discharged patients were advised to attend orthopaedic clinics. The patient who died had practically complete respiratory paralysis ; for twenty days she did fairly well in the mechanical respirator, and seemed to be improving. Then quite suddenly she developed acute dilatation of the stomach and died within twelve hours.

*Comment.* It is felt that the diagnosis of poliomyelitis rests fairly satisfactorily on the presence of typical paralysis, or cerebro-spinal fluid changes, or on both ; but if these things are not demonstrable the clinician is undoubtedly at a loss. He may perhaps consider contiguity with another proved case sufficient basis to call a doubtful illness poliomyelitis, but in the absence of laboratory proof of infection minor cases must necessarily be missed. This was well realized at Ham Green Hospital, but the preference for a diagnosis of poliomyelitis was not over-weighted by the presence of epidemic conditions. For example, it seemed wise to recognize "tonsillitis" in the case of a patient complaining of sore throat, headache and some stiffness of the neck, if inflammation of the fauces and cervical adenitis was noted in the absence of alteration of the cerebro-spinal fluid. Undoubtedly such a case would be classified tonsillitis in normal times.

The differential diagnosis of poliomyelitis in all its phases comprises a long list and therefore the notified cases far outnumbered the confirmed. In the cases considered here the relative figures are 73:18. The large figure indicates the comprehensive diagnostic sweep taken by practitioners, and is to be commended. If the revised diagnoses are considered it is seen that certain groups of symptoms conditioned the original diagnosis of poliomyelitis. These symptom groups were chiefly represented by (*a*) sore throat (twenty cases of tonsillitis), (*b*) meningeal syndrome (5 cases of meningococcal meningitis, one each of pneumococcal, influenzal, tuberculous and coliform meningitis, two of chorio-meningitis and two of subarachnoid haemorrhage), and (*c*) muscle and joint pains (three cases of acute rheumatism, six of influenzal colds, one of myalgia and one of osteomyelitis). In addition a variety of patients produced symptoms referable to the central nervous system, which might have simulated some phase of poliomyelitis; viz.—one case each of lateral sinus thrombosis, Bell's palsy, Landry's paralysis, brain abscess, epilepsy, meningism due to pneumonia, and periarteritis nodosa.

Rather more difficult to associate with a diagnosis of poliomyelitis were cases of pneumonia, infectious mono-nucleosis, menopause, myeloid leucaemia, pyelitis of pregnancy, psychosis, scarlatina-septic sores, axillary adenitis and otitis media. Four cases were considered entirely negative. Probably the most difficult diagnostic decision among all these cases was that of chorio-meningitis. This rather ill defined disease can easily be confused with abortive poliomyelitis.

In conclusion it was noted that only five of the twenty-two proved cases complained of sore throat, and no case had had recent surgical interference with the upper respiratory passages. On the other hand, whether significant or not, two cases of true poliomyelitis developed in the midst of an attack of scarlatina.

Sincere thanks are due to Dr. B. A. Peters, Superintendent of Ham Green Hospital, for his enthusiastic advice and assistance in dealing with the cases, and for permission to publish these notes.

